# Comparison of Symbiodiniaceae diversities in different members of a *Palythoa* species complex (Cnidaria: Anthozoa: Zoantharia)—implications for ecological adaptations to different microhabitats

**DOI:** 10.7717/peerj.8449

**Published:** 2020-02-03

**Authors:** Masaru Mizuyama, Akira Iguchi, Mariko Iijima, Kodai Gibu, James Davis Reimer

**Affiliations:** 1 Molecular Invertebrate Systematics and Ecology Laboratory, Graduate School of Engineering and Science, University of the Ryukyus, Nishihara, Okinawa, Japan; 2 Geological Survey of Japan, National Institute of Advanced Industrial Science and Technology (AIST), Tsukuba, Ibaraki, Japan; 3 Department of Bioresources Engineering, National Institute of Technology, Okinawa College, Nago, Okinawa, Japan; 4 Tropical Biosphere Research Center, University of the Ryukyus, Nishihara, Okinawa, Japan

**Keywords:** Zoantharia, *Palythoa* species complex, Symbiodiniaceae, Ecological divergence

## Abstract

In this study we compared genotypes of zoantharian host-associating algal symbionts among *Palythoa* species, which are among the dominant benthic reef organisms in the Ryukyu Archipelago, Japan, and evaluated Symbiodiniaceae diversities of closely related congeneric *Palythoa* species. We targeted a species complex of the zoantharian genus *Palythoa* (*P. tuberculosa*, *P.* sp. yoron, *P. mutuki*) living among different microhabitats in a narrow reef area of Tokunoshima Island. For phylogenetic analyses, we used two DNA marker regions; nuclear internal transcribed spacer (ITS) and plastid mini-circle non-coding region (psbA^ncr^), both of which have previously been used to determine Symbiodiniaceae genotypes of zoantharian species. Our results showed that all *Palythoa* species hosted symbionts of the genus *Cladocopium*, with genotypic compositions of this genus showing some variations among the three different *Palythoa* species. Additionally, we found that the *Cladocopium* genotypic composition was statistically different among *Palythoa* species, and among *P. tuberculosa* specimens in different microhabitats. Our results suggest that ecological divergence among these three *Palythoa* species may be related to differing Symbiodiniaceae diversities that may in turn contribute to eco-physiological adaptation into different microhabitats on coral reefs.

## Introduction

Zoantharians (Anthozoa: Zoantharia) belong to the phylum Cnidaria and can be dominant organisms in shallow coral reef areas (e.g.,  Burnett et al., 1994). In particular, the genus *Palythoa* is often among the most dominant benthos in coral reef areas (Irei, Nozawa & Reimer, 2011; Santos et al., 2016; Reimer et al., 2017a).

We recently reported on four putative *Palythoa* species (*P. tuberculosa*, *P.* sp. yoron, *P. mutuki*, and *P.* aff. *mutuki*) that form a species complex, and were observed to all occur within a narrow range of coral reefs in southern Japan (Mizuyama, Masucci & Reimer, 2018). For example, *P. tuberculosa* tends to occur across a wide range of habitats from shallow to deeper areas, from the intertidal zone to the mesophotic reef slope (Mizuyama, Masucci & Reimer, 2018), and has been reported from tropical to temperate regions (Reimer, Takishita & Maruyama, 2006). On the other hand, the other three *Palythoa* species appear to more restricted compared to *P. tuberculosa* in terms of their distribution and habitats within coral reefs. *Palythoa mutuki* is the second most dominant species in this genus in Okinawa and is often dominant at the reef edge, in surge channels, and in small bumps on reef flats (Irei, Nozawa & Reimer, 2011). *Palythoa* sp. yoron has yet to be formally described, but tends to occur on reef flats and backreef moats where it is exposed to strong water currents (Shiroma & Reimer, 2010). Although there is little published information on *P*. aff. *mutuki*, it has been observed near *P. mutuki* colonies on the reef flat (Mizuyama, Masucci & Reimer, 2018). Although molecular delineation of these *Palythoa* species groups was unsuccessful with molecular data, likely due to incomplete lineage sorting, they can be distinguished via morphological and reproductive data (Mizuyama, Masucci & Reimer, 2018). In addition, these *Palythoa* species display different microhabitat patterns within the coral reef, but it is still unclear how these species would have diversified under almost completely sympatric conditions.

Symbiodiniaceae endosymbiotic dinoflagellates are symbiotic with various metazoan phyla including Cnidaria (LaJeunesse et al., 2018). Many zoantharians maintain Symbiodiniaceae, similar to reef-building corals (Noda et al., 2017; Wee, Kurihara & Reimer, 2019). In the case of scleractinian corals, symbiotic relationships with Symbiodiniaceae are important for host survival in various environments (Baker, 2003), and can contribute to ecological divergence of coral host species (Winters et al., 2009). Previous molecular studies have reported that species composition of Symbiodiniaceae is closely related to host genotypes in corals (e.g.,  Bongaerts et al., 2010; Pinzon & LaJeunesse, 2011). Thus, information on the composition Symbiodiniaceae of the four *Palythoa* species above would also be helpful to understand their ecological divergence into different microenvironments within a reef. In particular, genotypic composition of symbiotic algae would be informative for understanding ecological divergence of these species because the genetic and/or community changes of microbiomes are expected to be faster than that of the hosts themselves (Torda et al., 2017), facilitating eco-physiological adaptation of holobionts into different microenvironments (e.g.,  Reimer et al., 2017b; Wee, Kurihara & Reimer, 2019). In this study, we aimed to (1) compare diversities of symbionts among the closely related *Palythoa* species *P. tuberculosa*, *P*. sp. yoron, *P. mutuki* and *P*. aff. *mutuki*, and (2) determine if diversities of symbionts explain eco-physiological adaptations to microhabitats of each species that entailed divergences among them (*P. tuberculosa*, *P*. sp. yoron and *P. mutuki*).

## Materials & Methods

### Specimens collection

**Figure 1:**
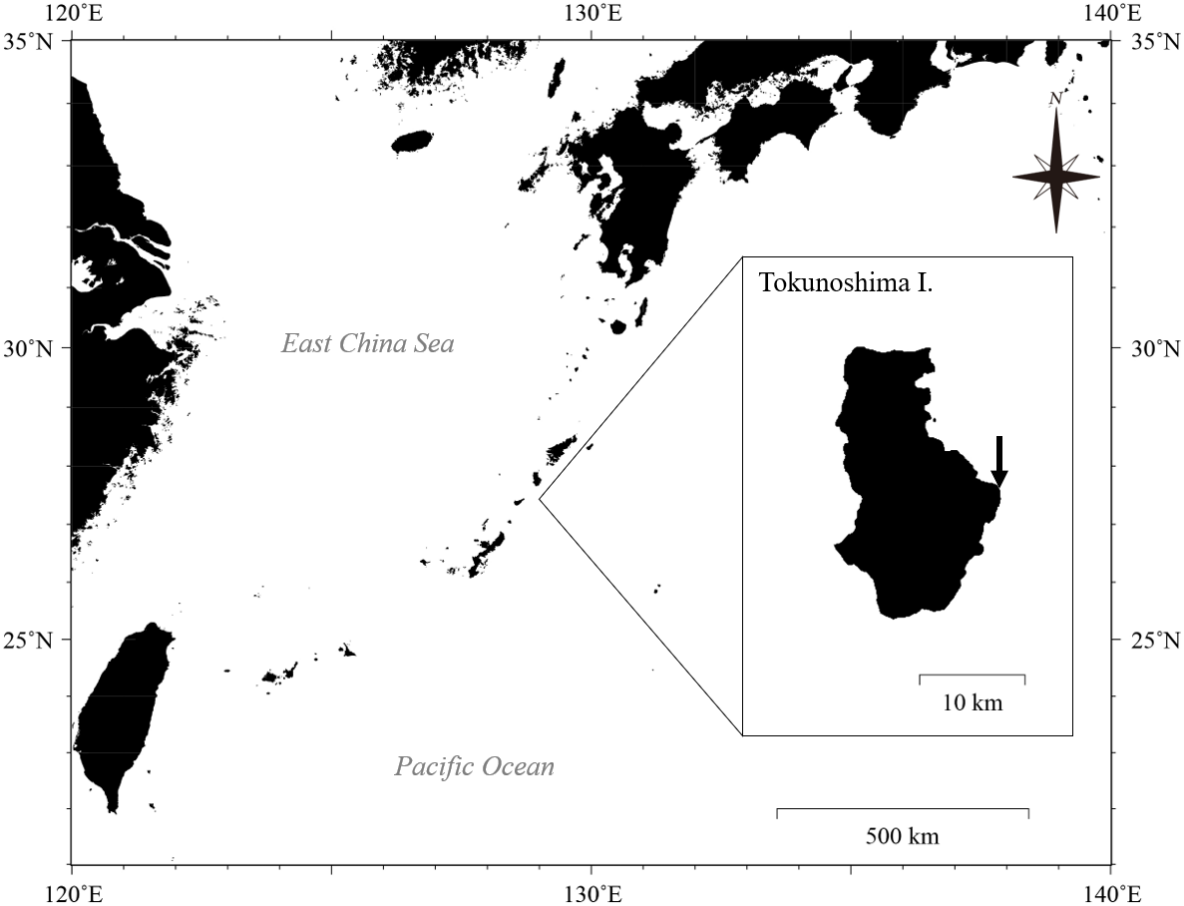
Location of Tokunoshima Island and the sampling site (arrow in inset) for the *Palythoa* specimens in this study. Map data: GeoLite2 data created by MaxMind using the Generic Mapping Tools (GMT v5.4.5) software package. CC BY SA 4.0.

**Figure 2:**
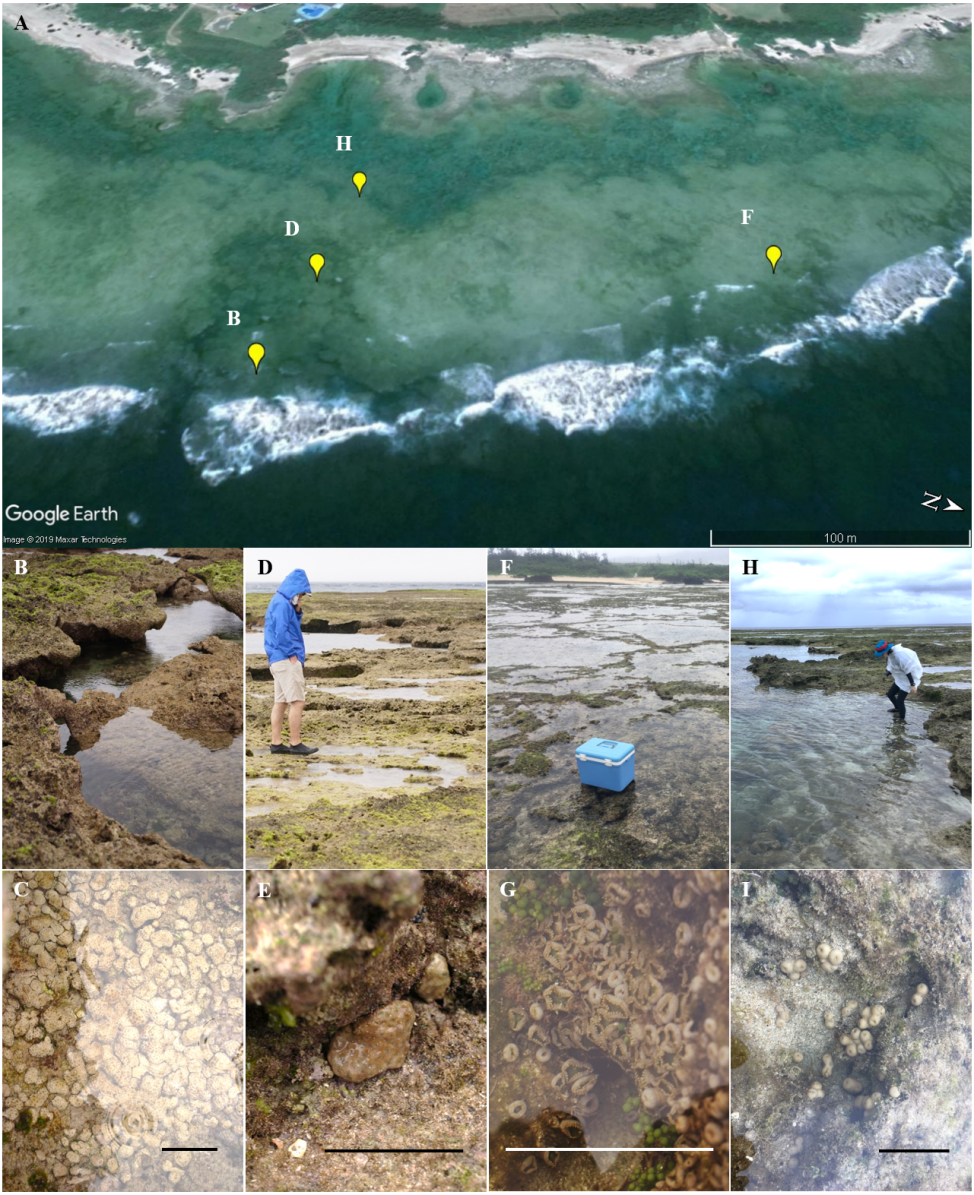
Landscape of the coral reef flat at the study site and in situ images of *Palythoa* species used in this study. (A) Satellite image of the reef area obtained by Google Earth; (B) reef edge; (C) *P. tubeculosa*; (D) reef flat 1; (E) *P. tuberculosa*; (F) reef flat 2; (G) *P. mutuki*; (H) backreef moat; (I) *P.* sp. yoron. Map data: Google, Maxar Technologies. Scale bars in C, E, G, and I are 10 cm.

Eighty-two colonies of three *Palythoa* species (*P. tuberculosa*, *P.* sp. yoron, and *P. mutuki*) were collected from a shallow fringing reef of Tokunoshima Island, Kagoshima, Japan (Figs. 1 and 2). Specimens of these three *Palythoa* species were collected in four different areas (Table 1, Fig. 2A): reef edge (Fig. 2B, 27.76998333N, 129.03988611E) for *P. tuberculosa* (Fig. 2C); reef flat 1 (Fig. 2D, 27.76997777N, 129.03925000E) for *P. tuberculosa* (Fig. 2E) and *P. mutuki*; reef flat 2 (Fig. 2F, 27.77195277N, 129.03843611E) for *P. mutuki* (Fig. 2G); and backreef moat (Fig. 2H, 27.76990833N, 129.03855833E) for *P. tuberculosa* and *P*. sp. yoron (Fig. 2I). To avoid collecting clones, we collected individuals from clearly different colonies while maintaining a set distance from each other of at least 1 m. In a previous study, even when closer to each other (within approximately 50 × 50 cm), no clones were observed in *Zoanthus* (Cnidaria: Anthozoa: Zoantharia) colonies (Albinsky et al., 2018). In addition, eighteen previously collected specimens of *Palythoa* species including 10 *P.* aff. *mutuki* specimens from Mizuyama, Masucci & Reimer (2018) were also examined in this study (Table 1).

### DNA extraction and PCR amplification

From each of these specimens, several polyps were cut with a surgical knife and DNA was extracted using DNeasy Blood and Tissue Kit (QIAGEN). DNA concentrations were checked by Qubit Fluorometer (ThermoFisher, Waltham, USA). Two molecular markers for genotyping symbiotic algae of *Palythoa* species were examined: nuclear internal transcribed spacer ribosomal DNA (ITS-rDNA) region including partial 18S–ITS1–5.8S–ITS2–partial 28S (primers zITSf: CCG GTG AAT TAT TCG GAC TGA CGC AGT and ITS4: TCC TCC GCT TAT TGA TAT GC, (Baillie, Belda-Baillie & Maruyama, 2000; appx. 700–750 bp) and plastid mini-circle non-coding region DNA (psbA^ncr^) (primers 7.4-Forw: GCA TGA AAG AAA TGC ACA CAA CTT CCC and 7.8-Rev: GGT TCT CTT ATT CCA TCA ATA TCT ACT G, (Moore et al., 2003; appx. 800–850 bp). These regions were amplified according to the PCR thermal conditions in Wee, Kurihara & Reimer (2019). Amplified PCR products of symbionts were directly sequenced, and sequence data were manually checked based on the chromatogram files and low quality sites were removed at the 5′ and 3′ ends by BioEdit v.7.0.5.3 (Hall, 1999). Obtained sequences were deposited in the GenBank database (MN654128–MN654306, Table 1).

**Table 1:** Specimen list.

Specimen ID	Location/Region	Spiecies ID	Date (m/d/y)	Environment	Accession no. of ITS	Accession no. of psbA-F	Accession no. of psbA-R
A01PtToKa	Kaminomine/Tokunoshima	*Palythoa tuberculosa*	Jun 2, 2019	Reef edge	MN654209	MN654185	–
A02PtToKa	Kaminomine/Tokunoshima	*Palythoa tuberculosa*	Jun 2, 2019	Reef edge	MN654210	MN654184	MN654134
A03PtToKa	Kaminomine/Tokunoshima	*Palythoa tuberculosa*	Jun 2, 2019	Reef edge	MN654211	–	–
A04PtToKa	Kaminomine/Tokunoshima	*Palythoa tuberculosa*	Jun 2, 2019	Reef edge	MN654212	MN654186	MN654135
A05PtToKa	Kaminomine/Tokunoshima	*Palythoa tuberculosa*	Jun 2, 2019	Reef edge	MN654213	MN654187	MN654136
A06PtToKa	Kaminomine/Tokunoshima	*Palythoa tuberculosa*	Jun 2, 2019	Reef edge	MN654214	MN654188	–
A07PtToKa	Kaminomine/Tokunoshima	*Palythoa tuberculosa*	Jun 2, 2019	Reef edge	MN654215	MN654189	MN654137
A08PtToKa	Kaminomine/Tokunoshima	*Palythoa tuberculosa*	Jun 2, 2019	Reef edge	MN654216	MN654190	MN654138
A09PtToKa	Kaminomine/Tokunoshima	*Palythoa tuberculosa*	Jun 2, 2019	Reef edge	MN654217	–	–
A11PtToKa	Kaminomine/Tokunoshima	*Palythoa tuberculosa*	Jun 2, 2019	Reef flat	MN654218	MN654191	MN654139
A12PtToKa	Kaminomine/Tokunoshima	*Palythoa tuberculosa*	Jun 2, 2019	Reef flat	MN654219	MN654192	MN654140
A13PtToKa	Kaminomine/Tokunoshima	*Palythoa tuberculosa*	Jun 2, 2019	Reef flat	MN654220	MN654193	–
A14PtToKa	Kaminomine/Tokunoshima	*Palythoa tuberculosa*	Jun 2, 2019	Reef flat	MN654221	–	–
A15PtToKa	Kaminomine/Tokunoshima	*Palythoa tuberculosa*	Jun 2, 2019	Reef flat	MN654222	–	–
A16PtToKa	Kaminomine/Tokunoshima	*Palythoa tuberculosa*	Jun 2, 2019	Reef flat	MN654223	MN654194	MN654141
A17PtToKa	Kaminomine/Tokunoshima	*Palythoa tuberculosa*	Jun 2, 2019	Reef flat	MN654224	–	–
A18PtToKa	Kaminomine/Tokunoshima	*Palythoa tuberculosa*	Jun 2, 2019	Reef flat	MN654225	MN654195	MN654142
A19PtToKa	Kaminomine/Tokunoshima	*Palythoa tuberculosa*	Jun 2, 2019	Reef flat	MN654226	MN654198	–
A20PtToKa	Kaminomine/Tokunoshima	*Palythoa tuberculosa*	Jun 2, 2019	Reef flat	MN654227	–	–
A21PtToKa	Kaminomine/Tokunoshima	*Palythoa tuberculosa*	Jun 2, 2019	Backreef moat	MN654228	MN654169	MN654159
A22PtToKa	Kaminomine/Tokunoshima	*Palythoa tuberculosa*	Jun 2, 2019	Backreef moat	MN654229	–	–
A24PtToKa	Kaminomine/Tokunoshima	*Palythoa tuberculosa*	Jun 2, 2019	Backreef moat	MN654230	–	MN654143
A25PtToKa	Kaminomine/Tokunoshima	*Palythoa tuberculosa*	Jun 2, 2019	Backreef moat	MN654231	–	–
A26PtToKa	Kaminomine/Tokunoshima	*Palythoa tuberculosa*	Jun 2, 2019	Backreef moat	MN654232	–	–
A27PtToKa	Kaminomine/Tokunoshima	*Palythoa tuberculosa*	Jun 2, 2019	Backreef moat	MN654233	–	–
A28PtToKa	Kaminomine/Tokunoshima	*Palythoa tuberculosa*	Jun 2, 2019	Backreef moat	MN654234	–	–
A29PtToKa	Kaminomine/Tokunoshima	*Palythoa tuberculosa*	Jun 2, 2019	Backreef moat	MN654235	–	–
A30PtToKa	Kaminomine/Tokunoshima	*Palythoa tuberculosa*	Jun 2, 2019	Backreef moat	MN654236	–	–
B01PmToKa	Kaminomine/Tokunoshima	*Palythoa mutuki*	Jun 2, 2019	Reef flat	MN654237	–	–
B02PmToKa	Kaminomine/Tokunoshima	*Palythoa mutuki*	Jun 2, 2019	Reef flat	MN654238	MN654199	MN654144
B03PmToKa	Kaminomine/Tokunoshima	*Palythoa mutuki*	Jun 2, 2019	Reef flat	MN654239	–	–
B04PmToKa	Kaminomine/Tokunoshima	*Palythoa mutuki*	Jun 2, 2019	Reef flat	MN654240	–	–
B05PmToKa	Kaminomine/Tokunoshima	*Palythoa mutuki*	Jun 2, 2019	Reef flat	MN654241	–	MN654145
B06PmToKa	Kaminomine/Tokunoshima	*Palythoa mutuki*	Jun 2, 2019	Reef flat	MN654242	MN654170	MN654160
B07PmToKa	Kaminomine/Tokunoshima	*Palythoa mutuki*	Jun 2, 2019	Reef flat	MN654243	–	MN654161
B08PmToKa	Kaminomine/Tokunoshima	*Palythoa mutuki*	Jun 2, 2019	Reef flat	MN654244	MN654171	MN654162
B09PmToKa	Kaminomine/Tokunoshima	*Palythoa mutuki*	Jun 2, 2019	Reef flat	MN654245	–	–
B11PmToKa	Kaminomine/Tokunoshima	*Palythoa mutuki*	Jun 2, 2019	Reef flat	MN654246	–	–
B12PmToKa	Kaminomine/Tokunoshima	*Palythoa mutuki*	Jun 3, 2019	Reef flat	MN654247	MN654172	–
B13PmToKa	Kaminomine/Tokunoshima	*Palythoa mutuki*	Jun 3, 2019	Reef flat	MN654248	–	–
B14PmToKa	Kaminomine/Tokunoshima	*Palythoa mutuki*	Jun 3, 2019	Reef flat	MN654249	MN654173	–
B15PmToKa	Kaminomine/Tokunoshima	*Palythoa mutuki*	Jun 3, 2019	Reef flat	MN654250	–	–
B16PmToKa	Kaminomine/Tokunoshima	*Palythoa mutuki*	Jun 3, 2019	Reef flat	MN654251	–	–
B17PmToKa	Kaminomine/Tokunoshima	*Palythoa mutuki*	Jun 3, 2019	Reef flat	MN654252	MN654174	MN654163
B18PmToKa	Kaminomine/Tokunoshima	*Palythoa mutuki*	Jun 3, 2019	Reef flat	MN654253	MN654200	–
B20PmToKa	Kaminomine/Tokunoshima	*Palythoa mutuki*	Jun 3, 2019	Reef flat	MN654254	–	–
B21PmToKa	Kaminomine/Tokunoshima	*Palythoa mutuki*	Jun 3, 2019	Reef flat	MN654255	–	–
B22PmToKa	Kaminomine/Tokunoshima	*Palythoa mutuki*	Jun 3, 2019	Reef flat	MN654256	–	–
B23PmToKa	Kaminomine/Tokunoshima	*Palythoa mutuki*	Jun 3, 2019	Reef flat	MN654257	–	–
B24PmToKa	Kaminomine/Tokunoshima	*Palythoa mutuki*	Jun 3, 2019	Reef flat	MN654258	MN654175	MN654164
B25PmToKa	Kaminomine/Tokunoshima	*Palythoa mutuki*	Jun 3, 2019	Reef flat	–	MN654176	MN654165
B26PmToKa	Kaminomine/Tokunoshima	*Palythoa mutuki*	Jun 3, 2019	Reef flat	MN654259	–	MN654166
B28PmToKa	Kaminomine/Tokunoshima	*Palythoa mutuki*	Jun 3, 2019	Reef flat	–	MN654177	MN654167
C01PyToKa	Kaminomine/Tokunoshima	*Palythoa* sp. yoron	Jun 2, 2019	Backreef moat	MN654260	–	–
C02PyToKa	Kaminomine/Tokunoshima	*Palythoa* sp. yoron	Jun 2, 2019	Backreef moat	MN654261	–	–
C03PyToKa	Kaminomine/Tokunoshima	*Palythoa* sp. yoron	Jun 2, 2019	Backreef moat	MN654262	–	–
C04PyToKa	Kaminomine/Tokunoshima	*Palythoa* sp. yoron	Jun 2, 2019	Backreef moat	MN654263	–	–
C05PyToKa	Kaminomine/Tokunoshima	*Palythoa* sp. yoron	Jun 2, 2019	Backreef moat	MN654264	–	–
C06PyToKa	Kaminomine/Tokunoshima	*Palythoa* sp. yoron	Jun 2, 2019	Backreef moat	MN654265	–	–
C07PyToKa	Kaminomine/Tokunoshima	*Palythoa* sp. yoron	Jun 2, 2019	Backreef moat	MN654266	–	–
C08PyToKa	Kaminomine/Tokunoshima	*Palythoa* sp. yoron	Jun 2, 2019	Backreef moat	MN654267	–	–
C09PyToKa	Kaminomine/Tokunoshima	*Palythoa* sp. yoron	Jun 2, 2019	Backreef moat	MN654268	–	–
C10PyToKa	Kaminomine/Tokunoshima	*Palythoa* sp. yoron	Jun 2, 2019	Backreef moat	MN654269	–	–
C11PyToKa	Kaminomine/Tokunoshima	*Palythoa* sp. yoron	Jun 2, 2019	Backreef moat	MN654270	–	–
C12PyToKa	Kaminomine/Tokunoshima	*Palythoa* sp. yoron	Jun 2, 2019	Backreef moat	MN654271	MN654201	MN654146
C13PyToKa	Kaminomine/Tokunoshima	*Palythoa* sp. yoron	Jun 2, 2019	Backreef moat	MN654272	–	–
C14PyToKa	Kaminomine/Tokunoshima	*Palythoa* sp. yoron	Jun 2, 2019	Backreef moat	MN654273	MN654179	MN654147
C15PyToKa	Kaminomine/Tokunoshima	*Palythoa* sp. yoron	Jun 2, 2019	Backreef moat	MN654274	MN654180	–
C16PyToKa	Kaminomine/Tokunoshima	*Palythoa* sp. yoron	Jun 2, 2019	Backreef moat	MN654275	MN654202	MN654148
C17PyToKa	Kaminomine/Tokunoshima	*Palythoa* sp. yoron	Jun 2, 2019	Backreef moat	MN654276	MN654203	MN654149
C18PyToKa	Kaminomine/Tokunoshima	*Palythoa* sp. yoron	Jun 2, 2019	Backreef moat	MN654277	–	MN654168
C19PyToKa	Kaminomine/Tokunoshima	*Palythoa* sp. yoron	Jun 3, 2019	Backreef moat	MN654278	–	–
C20PyToKa	Kaminomine/Tokunoshima	*Palythoa* sp. yoron	Jun 3, 2019	Backreef moat	MN654279	MN654204	MN654150
C21PyToKa	Kaminomine/Tokunoshima	*Palythoa* sp. yoron	Jun 3, 2019	Backreef moat	MN654280	MN654205	MN654151
C22PyToKa	Kaminomine/Tokunoshima	*Palythoa* sp. yoron	Jun 3, 2019	Backreef moat	MN654281	MN654206	MN654152
C24PyToKa	Kaminomine/Tokunoshima	*Palythoa* sp. yoron	Jun 3, 2019	Backreef moat	MN654282	MN654181	–
C25PyToKa	Kaminomine/Tokunoshima	*Palythoa* sp. yoron	Jun 3, 2019	Backreef moat	MN654283	MN654196	MN654153
C26PyToKa	Kaminomine/Tokunoshima	*Palythoa* sp. yoron	Jun 3, 2019	Backreef moat	MN654284	–	MN654154
C27PyToKa	Kaminomine/Tokunoshima	*Palythoa* sp. yoron	Jun 3, 2019	Backreef moat	MN654285	MN654207	MN654155
C28PyToKa	Kaminomine/Tokunoshima	*Palythoa* sp. yoron	Jun 3, 2019	Backreef moat	MN654286	–	–
C29PyToKa	Kaminomine/Tokunoshima	*Palythoa* sp. yoron	Jun 3, 2019	Backreef moat	MN654287	MN654208	MN654156
C30PyToKa	Kaminomine/Tokunoshima	*Palythoa* sp. yoron	Jun 3, 2019	Backreef moat	MN654288	MN654182	MN654157
159PamToKa	Kaminomine/Tokunoshima	*Palythoa* aff. *mutuki*	July 28, 2010	In Mizuyama, Masucci & Reimer (2018)	MN654300	–	–
233PamErYa	Yakomo/Okinoerabu	*Palythoa* aff. *mutuki*	Jun 17, 2011	In Mizuyama, Masucci & Reimer (2018)	MN654301	–	–
237PamErSu	Sumiyoshi/Okinoerabu	*Palythoa* aff. *mutuki*	Jun 18, 2011	In Mizuyama, Masucci & Reimer (2018)	MN654302	–	–
248PamToKa	Kaminomine/Tokunoshima	*Palythoa* aff. *mutuki*	Jun 21, 2011	In Mizuyama, Masucci & Reimer (2018)	MN654303	–	–
250PamToKa	Kaminomine/Tokunoshima	*Palythoa* aff. *mutuki*	Jun 21, 2011	In Mizuyama, Masucci & Reimer (2018)	MN654304	MN654183	MN654131
328PamOkTe	Teniya/Okinawa	*Palythoa* aff. *mutuki*	Apr 5, 2012	In Mizuyama, Masucci & Reimer (2018)	MN654305	–	–
364PamOkOk	Oku/Okinawa	*Palythoa* aff. *mutuki*	Jun 25, 2012	In Mizuyama, Masucci & Reimer (2018)	MN654306	–	–
2PtOkOd	Odo/Okinawa	*Palythoa tuberculosa*	Aug 18, 2009	In Mizuyama, Masucci & Reimer (2018)	MN654289	–	MN654158
39PtYoUk	Ukachi/Yoron	*Palythoa tuberculosa*	Mar 4, 2010	In Mizuyama, Masucci & Reimer (2018)	MN654290	–	MN654132
63PtErYa	Yakomo/Okinoerabu	*Palythoa tuberculosa*	Mar 5, 2010	In Mizuyama, Masucci & Reimer (2018)	MN654291	–	MN654133
100PtToKa	Kaminomine/Tokunoshima	*Palythoa tuberculosa*	Mar 9, 2010	In Mizuyama, Masucci & Reimer (2018)	MN654292	–	MN654128
15PyOkOd	Odo/Okinawa	*Palythoa* sp. yoron	Sep 5, 2009	In Mizuyama, Masucci & Reimer (2018)	MN654297	–	MN654130
51PyYoUk	Ukachi(West)/Yoron	*Palythoa* sp. yoron	Mar 4, 2010	In Mizuyama, Masucci & Reimer (2018)	MN654298	–	–
85PyErYa	Yakomo/Okinoerabu	*Palythoa* sp. yoron	Mar 5, 2010	In Mizuyama, Masucci & Reimer (2018)	MN654296	MN654197	–
105PyToKa	Kaminomine/Tokunoshima	*Palythoa* sp. yoron	Mar 9, 2010	In Mizuyama, Masucci & Reimer (2018)	MN654299	MN654178	MN654129
218PmOkOd	Odo/Okinawa	*Palythoa mutuki*	May 4, 2011	In Mizuyama, Masucci & Reimer (2018)	MN654294	–	–
77PmErYa	Yakomo/Okinoerabu	*Palythoa mutuki*	Mar 5, 2010	In Mizuyama, Masucci & Reimer (2018)	MN654293	–	–
280PmToKa	Kaminomine/Tokunoshima	*Palythoa mutuki*	Oct 5, 2011	In Mizuyama, Masucci & Reimer (2018)	MN654295	–	–

### Haplotype network inference and phylogenetic estimation

Obtained sequences for ITS-rDNA, psbA^ncr^ forward and reverse regions were aligned, respectively. In order to discriminate taxa of Symbiodiniaceae, we extracted the ITS2 region utilizing SymPortal (Hume et al., 2019; https://symportal.org/) and performed BLASTN search against the nt database using the NCBI website (https://blast.ncbi.nlm.nih.gov/Blast.cgi) for ITS-rDNA sequences. Haplotype network inference was performed for ITS-rDNA sequences using the alignment with TCS networks method (Clement et al., 2002) in PopART (Leigh & Bryant, 2015). Any columns in the alignment with gaps or ambiguous sites were automatically masked in the inference. The phylogenetic analyses were performed by MEGA version X (Kumar et al., 2018) and any loci with ambiguous (double peaks) sites and gaps was automatically deleted completely for calculation in order to avoid over/underestimation of genetic distance among each sequence. Molecular phylogenetic trees of each marker were constructed by maximum likelihood (ML) and neighbor joining (NJ) methods under the JC+G model for ITS-rDNA region and the JC model for psbA^ncr^ regions adopted by modeltest program within MEGA X. The significance of each node was tested by bootstrap test with 1,000 replications. Bayesian inference was performed using BEAST2 (Bouckaert et al., 2019) under default settings other than the clock model being changed to the relaxed log normal model, which showed the highest likelihood value according to the model comparison program compiled in BEAST2 (Drummond et al., 2006). Posterior probability (PP) on each branch was calculated summarizing four independent 10 million MCMC simulations.

### Statistical analyses

To clarify the relationships between (1) symbiont lineages and host species, and (2) symbiont lineages and host microhabitats, Fisher’s exact test was conducted for the compositions of genotype for ITS-rDNA region and monophyletic clades for psbA^ncr^ forward and reverse regions. It should be noted that host microhabitat was restricted by host species for *P*. sp. yoron and *P. mutuki*, and thus we only targeted *P. tuberculosa* for these analyses (aim 2 above) When significance was detected in Fisher’s exact test, Cramér’s coefficient of association (V) was calculated to evaluate which factors (host species or host microhabitat) were strongly associated with each other.

## Results

### Sequence alignment

The total number of sequences of Symbiodiniaceae from specimens of the four *Palythoa* species obtained in this study was 98 sequences for the ITS-rDNA region (513–773 bp), 40 sequences for the psbA^ncr^ forward region (330–547 bp), and 41 sequences for the psbA^ncr^ reverse region (352–494 bp). As the primer set for psbA^ncr^ used in this study did not make a congruent contig, obtained sequences of forward regions and reverse regions were aligned separately (Noda et al., 2017). After alignment, a total of 449 sites with 5 parsimony informative (=PI) sites for the ITS-rDNA region, 260 sites with 94 PI sites for the psbA^ncr^ forward region, and 293 sites with 40 PI sites for the psbA^ncr^ reverse region were used for each phylogenetic estimation.

### Barcoding, haplotype network and phylogenetic trees

**Figure 3:**
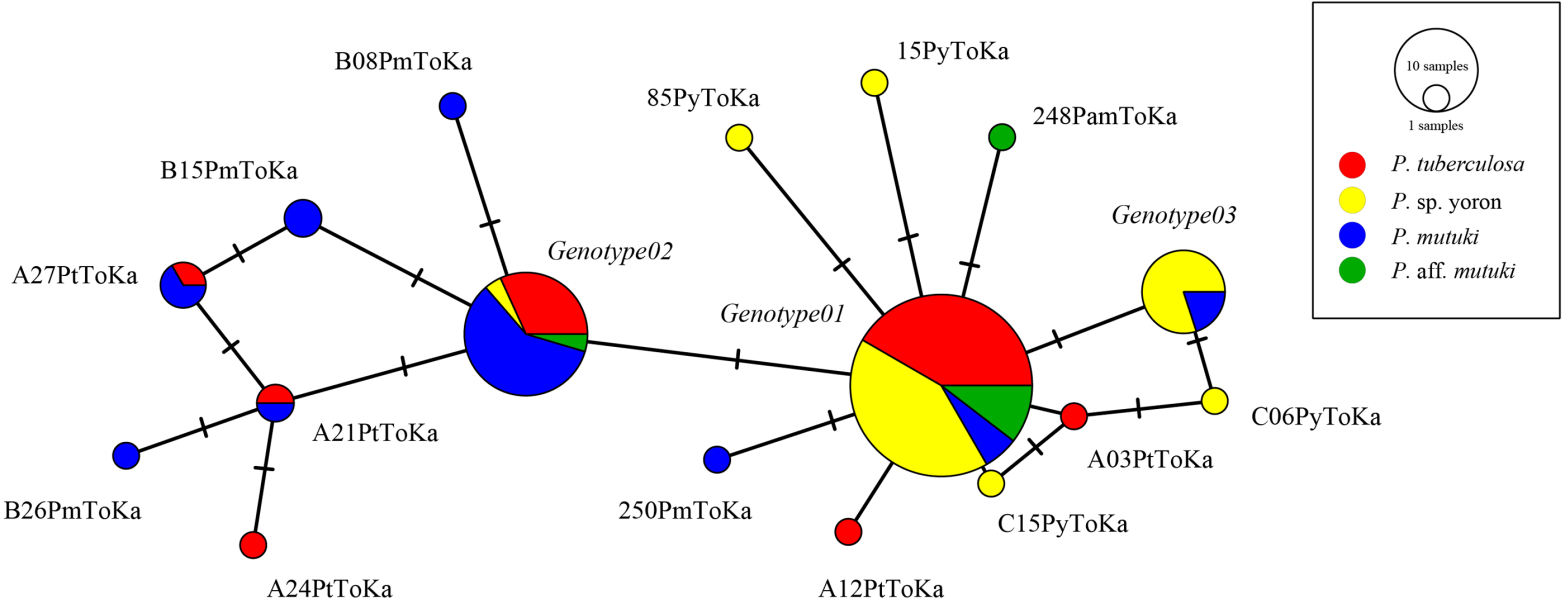
Haplotype network tree constructed with nuclear ITS-rDNA region alignment using TCS networks method. Scale represents number of sequences with circle sizes proportional to haplotype frequency. Colors represent *Palythoa* species: red, *P. tuberculosa*; yellow, *P*. sp. yoron; blue, *P. mutuki*; green, *P*. aff. *mutuki*.

As the result of BLAST searches, all query sequences of the ITS-rDNA region (*n* = 98) were confirmed as belonging to the genus *Cladocopium*. Seventeen ITS-rDNA unique sequences (=genotypes) were observed in TCS network, with most of the sequences belonging to one of major three ITS-rDNA genotypes (Fig. 3, Table S1). No significant clade was detected for the ITS-rDNA phylogenetic tree (Fig. S1). Summarizing these ITS-rDNA genotypes from the viewpoint of host species, *P. tuberculosa* possessed mainly *Genotype01* (*n* = 20) followed by *Genotype02* (*n* = 7), and *P*. sp. yoron also possessed mainly *Genotype01* (*n* = 20) followed by *Genotype03* (*n* = 8) (see details in Table S1). On the other hand, *P. mutuki* possessed mainly *Genotype02* (*n* = 13) with a few *Genotype01* (*n* = 3) and *Genotype03* (*n* = 2). Although the number of specimens examined was smaller (*n* = 6) than those the other species, *P.* aff. *mutuki* also possessed mainly *Genotype01* (*n* = 5).

**Figure 4:**
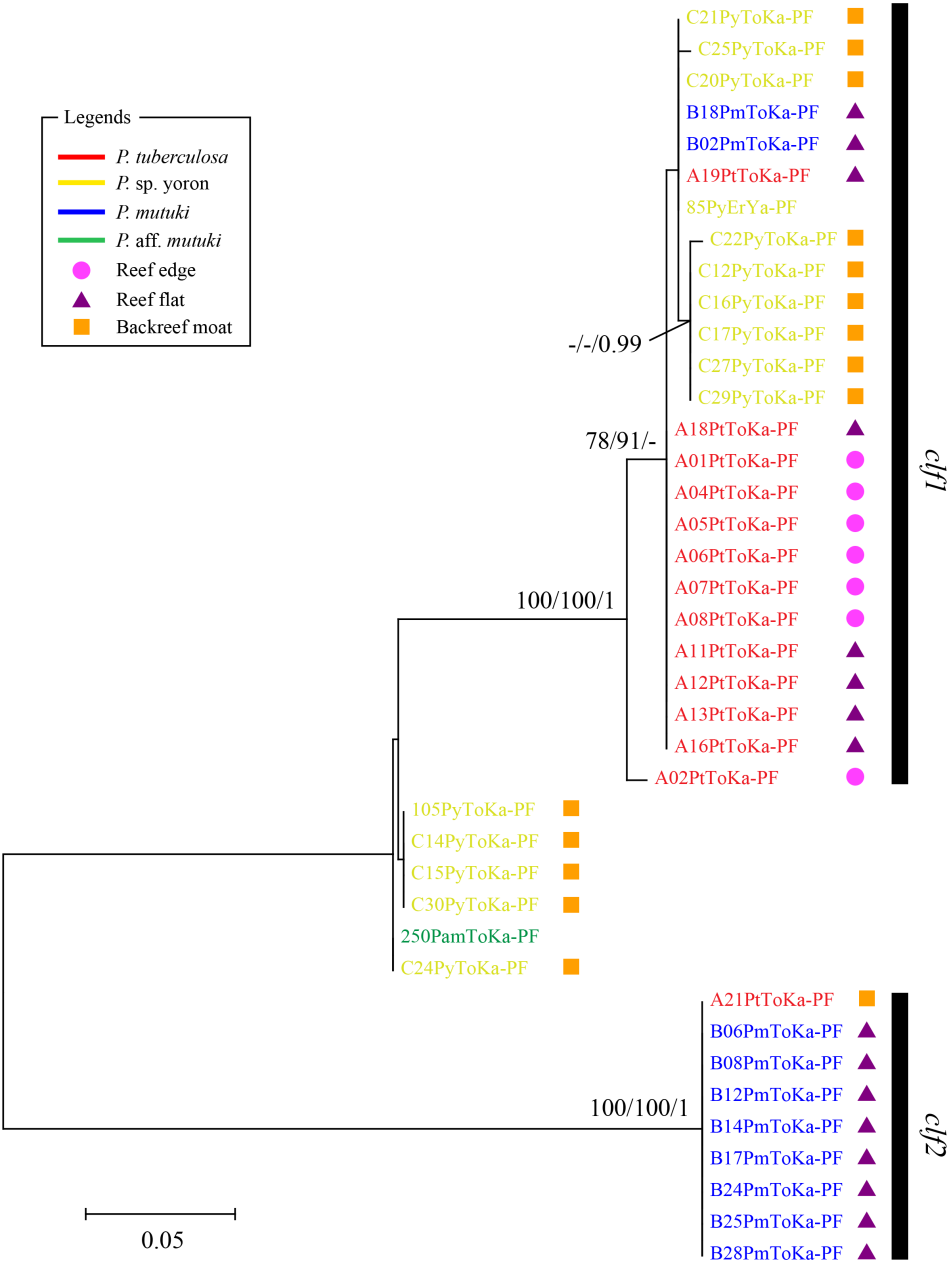
Molecular phylogenetic tree of Symbiodiniaceae of *Palythoa* species using mitochondrial psbA^ncr^ forward region. Bootstrap values of maximum likelihood (ML) and neighbor joining (NJ) methods, and posterior probability (PP) are shown more than 70% for ML and NJ, and more than 0.95 for PP at the nodes, respectively. Scale bars indicate substitutions per site. Colored letters and colored diagrams represent *Palythoa* species and their habitats, respectively: red, *P. tuberculosa*; yellow, *P*. sp. yoron; blue, *P. mutuki*; green, *P*. aff. *mutuki*; circle in pink, reef edge; triangle in purple, reef flat; square in orange, backreef moat.

**Figure 5:**
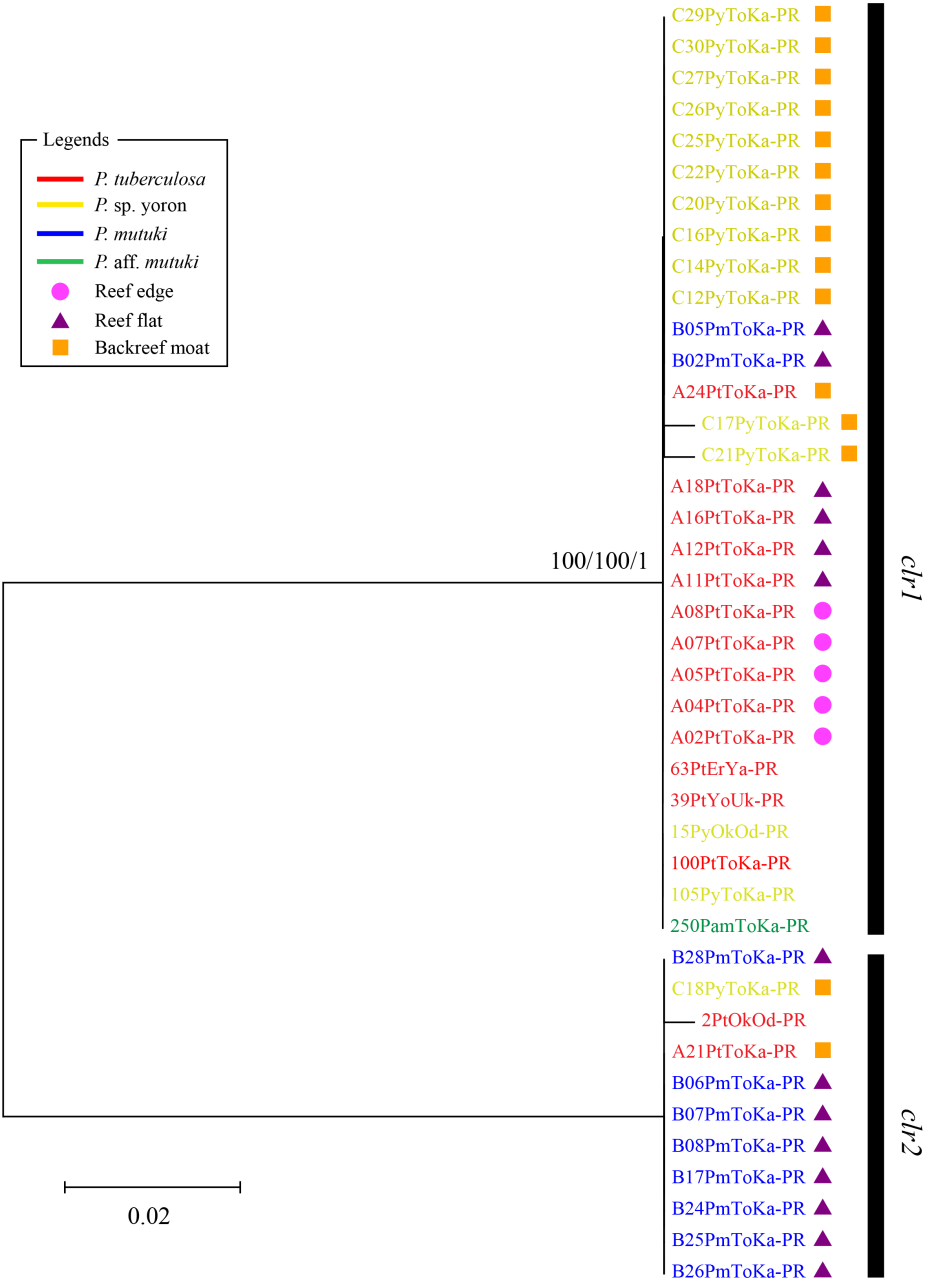
Molecular phylogenetic tree of Symbiodiniaceae of *Palythoa* species using mitochondrial psbA^ncr^ reverse region. Bootstrap values of maximum likelihood (ML) and neighbor joining (NJ) methods, and posterior probability (PP) are shown more than 70% for ML and NJ, and more than 0.95 for PP at the nodes, respectively. Scale bars indicate substitutions per site. Colored letters and colored diagrams represent *Palythoa* species and their habitats, respectively: red, *P. tuberculosa*; yellow, *P*. sp. yoron; blue, *P. mutuki*; green, *P*. aff. *mutuki*; circle in pink, reef edge; triangle in purple, reef flat; square in orange, backreef moat.

In contrast, phylogenetic trees generated from psbA^ncr^ regions had a higher resolution. Two monophyletic clades were well supported by bootstrap values and posterior probability in both forward (Fig. 4 *clf1*, ML = 100, NJ = 100, PP = 1 and *clf2*, ML = 100, NJ = 100, PP = 1) and reverse trees (Fig. 5 *clr1* and *clr2*, ML = 100, NJ = 100, PP = 1). Summarizing these Symbiodiniaceae lineages from the viewpoint of host species, *P. tuberculosa* inhabiting the reef edge possessed *clf1*/*clr1* lineage (*n* = 7∕5) and one specimen inhabiting at the backreef moat possessed *clf2*/*clr2* lineage. *Palythoa* sp. yoron inhabiting at the backreef moat possessed mainly *clf1*/*clr1* (*n* = 9∕13), however, approximately one third of specimens (*n* = 5) possessed other lineages*.* On the other hand, *P. mutuki* inhabiting the reef flat possessed mainly *clf2*/*clr2* (*n* = 8∕8) other than two specimens that possessed *clf1*/*clr1*. Unfortunately, as most of *P*. aff. *mutuki* specimens were not amplified by this primer set, we could only obtain phylogenetic information on one specimen which possessed the same lineage as *P*. sp. yoron (C24ToKa-PF) for the forward region and *clr1* for the reverse region.

### Relationships among symbiont genotype/lineages, host species and host microhabitats

From the results of Fisher’s Exact test, significant differences were detected in all combinations, i.e., ITS-rDNA genotype and host species (*p* < 0.01), psbA^ncr^ forward lineages and host species (*p* < 0.01), psbA^ncr^ reverse lineages and host species (*p* < 0.01), and ITS-rDNA genotype and host microhabitats for *P. tuberculosa* (*p* < 0.05) (Table 2). In other words, it was shown that Symbiodiniaceae lineages and host species were not independent, nor were Symbiodiniaceae lineages and host microhabitats for *P. tuberculosa*. The effective dose calculated by Cramér’s coefficient of association (V) was largest between host species and psbA^ncr^ forward/reverse lineages (*V* = 0.786, *V* = 0.682, respectively), and moderate for the other combinations (host species and ITS-rDNA genotypes, *V* = 0.477; host microhabitats and ITS-rDNA genotypes).

**Table 2:** Composition of genotype for ITS-rDNA sequences and monophyletic clades for psbA^ncr^ sequences of Symbiodiniaceae from *Palythoa* species used in this study and microenvironments of host habitats. Significances were tested by Fisher’s Exact Test and V value represents Cramer’s coefficient of association.

		Symbiodiniaceae genotype (ITS-rDNA)			Symbiodiniaceae lineage (psbA^ncr^ forward region)		Symbiodiniaceae lineage (psbA^ncr^ reverse region)	
		*Genotype01*	*Genotype02*	*Genotype03*	*clf1*	*clf2*	*clr1*	*clr2*
Host species	*P. tuberculosa*	20	7	0	13	1	13	2
	*P.* sp. yoron	20	1	8	10	0	14	1
	*P. mutuki*	3	13	2	2	8	2	8
	*P.* aff. *mutuki*	5	1	0	–	–	–	–
	Total	48	22	10	25	9	29	11
		*p* < 0.01, *V* = 0.477			*p* < 0.01, *V* = 0.786		*p* < 0.01, *V* = 0.682	
Host habitats of *P. tuberculosa*	Reef edge	8	0					
	Reef flat	7	2					
	Backreef moat	2	4					
	Total	17	6					
		*p* < 0.05, *V* = 0.508						

**Notes.**

*P*. aff. *mutuki* was removed from statistical analyses of psbA^ncr^ region due to low numbers of specimens.

## Discussion

### Symbiodiniaceae genotype/lineage and host species

The development of molecular markers such as psbA^ncr^ that have higher resolution than commonly used 18S or ITS ribosomal DNA markers has helped unveil a more detailed picture of the genetic diversity of Symbiodiniaceae (Takishita et al., 2003; LaJeunesse & Thornhill, 2011; LaJeunesse et al., 2018) (but see also Hume et al., 2019 who utilized intragenomic variation of ITS2 to resolve genetic delineations). Accordingly, host species biodiversity has been discovered from the initial observation of differences of Symbiodiniaceae phylotypes in some cnidarian species (e.g., gorgonian *Eunicea flexuosa*, Prada et al., 2014; scleractinian coral *Seriatopora hystrix*, Warner, Van Oppen & Willis, 2015).

From the results of Mizuyama, Masucci & Reimer (2018), none of the four molecular markers utilized could clearly delineate four *Palythoa* species, although they could delineate two closely related species groups composed of *P. tuberculosa*—*P.* sp. yoron and *P. mutuki*—*P*. aff. *mutuki*. These previous results seem to be reflected in the results in the current study of Symbiodiniaceae genotypes of ITS-rDNA and lineages of psbA^ncr^ regions. *Palythoa tuberculosa* and *P*. sp. yoron mostly shared the same symbiont genotype (*Genotype01*); nevertheless, they also partially shared the other genotypes with *P. mutuki* (*Genotype02* and *Genotype03*). With regard to psbA^ncr^ lineages, even though the delineation of species groups between *P. tuberculosa*—*P.* sp. yoron and *P. mutuki* were shown more clearly, they were not divided completely. The situation requires further investigation via obtaining more *P*. aff. *mutuki* specimens’ psbA^ncr^ sequences. Unfortunately, in the current study, despite much searching, we could not find large numbers of *P*. aff. *mutuki* on the reef in Tokunoshima Island, even though they were previous sampled for Mizuyama, Masucci & Reimer (2018). We do not know what happened to *P*. aff. *mutuki* colonies, but they may have been strongly affected by the bleaching events of 2016 and 2017 observed in southern Japan (Masucci et al., 2019).

### Symbiodiniaceae genotype/lineage and microhabitat of host species

From the results of the phylogenetic analyses, three microhabitats were not exclusively allocated in distinct Symbiodiniaceae genotypes or monophyletic clades, but the ratios of different genotypes were significantly different for *P. tuberculosa*. Regarding *P. tuberculosa*, Symbiodiniaceae *Genotype01* was mostly detected on the reef edge and reef flat, while *Genotype02* was mainly observed in the backreef moat. Although there were not enough samples to conduct statistical examinations of *P*. sp. yoron and *P. mutuki* due to their habitat specificity, *Genotype02* and *clf2*/*clr2* were detected mainly on the reef flat, while *Genotype01* and *clf1*/*clr1* were observed from all three environments.

It has been reported that zoantharian species with different symbiotic genotypes show species-specific photosynthetic responses against seawater temperature and *p* CO_2_ (Graham & Sanders, 2016; Reimer et al., 2017b; Wee, Kurihara & Reimer, 2019). Although the four *Palythoa* species in this study occurred sympatrically on one reef, the environmental conditions in a reef can be quite different according to small-scale geographical features. Seawater temperatures on reef flats frequently reach near 40 °C (Achituv & Dubinsky, 1990). In enclosed reefs, seawater temperatures and *p* CO_2_ show higher variations than those in exposed reefs (Suzuki, Nakamori & Kayanne, 1995; Fitt et al., 2001). Thus, the relationship between Symbiodiniaceae and host *Palythoa* species may change among different microhabitats in a reef area, facilitating ecological divergence of *Palythoa* species within a narrow geographic range.

Although a previous molecular study could not distinguish the boundaries among these *Palythoa* species (Mizuyama, Masucci & Reimer, 2018), it is suggested by our results that these species are ecologically divergent, and physiological differences within Symbiodiniaceae species may contribute to their ecological adaptation. In fact, Howells et al. (2012) reported that *Cladocopium* C1 in *Acropora tenuis* showed different physiological responses between northern and southern populations in the Great Barrier Reef. Considering that *Cladocopium* contains various species distinguished by differences of only a few bp in the ITS2 maker (Thornhill et al., 2014), meta-barcoding analyses via next-generation sequencing would be necessary to further understand the detailed relationship between Symbiodiniaceae and *Palythoa* species complex.

## Conclusions

We succeeded in obtaining genotypic data of Symbiodiniaceae from four putative *Palythoa* species and detected micro-scale geographic variations of the symbiotic algae among these species within a single coral reef. Our results suggest that ecological divergence among *Palythoa* species may be related to differences in Symbiodiniaceae diversities among microhabitats, even within a narrow reef area. More powerful genetic data such as that generated by next-generation sequencing could provide us with additional understanding on how neighboring *Palythoa* species have co-evolved with Symbiodiniaceae among the different microhabitats in a reef.

##  Supplemental Information

10.7717/peerj.8449/supp-1 Table S1 Composition of genotypes for ITS-rDNA sequences of Simbiodiniaceae from 4 *Palythoa* species

10.7717/peerj.8449/supp-2 Figure S1 Molecular phylogenetic tree of Symbiodiniaceae of *Palythoa* species using sequences of the nuclear ITS-rDNA region Shaded boxes represent three main genotypes occupying most sequences from four *Palythoa* species. Bootstrap values of maximum likelihood (ML) and neighbor joining (NJ) methods, and posterior probability (PP) are shown more than 70% for ML and NJ, and more than 0.95 for PP at the nodes, respectively. Scale bars indicate substitutions per site. Colored letters and colored diagrams represent *Palythoa* species and their habitats, respectively: red, *P. tuberculosa*; yellow, *P*. sp. yoron; blue, *P. mutuki*; green, *P*. aff. *mutuki*; circle in pink, reef edge; triangle in purple, reef flat; square in orange, backreef moat.

10.7717/peerj.8449/supp-3 Supplemental Information 3 The alignment files of sequences for ITS-rDNA, 98 sequences; psbA^ncr^ forward region, 40 sequences; psbA^ncr^ reverse region, 41 sequences

10.7717/peerj.8449/supp-4 Supplemental Information 4 The chromatogram files for ITS-rDNA region (forward)

10.7717/peerj.8449/supp-5 Supplemental Information 5 The chromatogram files for ITS-rDNA region (reverse)

10.7717/peerj.8449/supp-6 Supplemental Information 6 The chromatogram files for psbA^ncr^ forward region

10.7717/peerj.8449/supp-7 Supplemental Information 7 The chromatogram files for psbA^ncr^ reverse region

## Additional Information and Declarations

### Field Study Permissions

The following information was supplied relating to field study approvals (i.e., approving body and any reference numbers): No permission or institutional approval is required for collecting Zoantharia in Japan. Please see Mizuyama, Masucci & Reimer, 2018 or contact James D. Reimer for details (University of the Ryukyus; email: jreimer@sci.u-ryukyu.ac.jp).

### DNA Deposition

The following information was supplied regarding the deposition of DNA sequences: Nuclear internal transcribed spacer ribosomal DNA (ITS-rDNA) and plastid mini-circle non-coding region (psbAncr) sequences of Symbiodiniaceae are available at GenBank: MN654128–MN654306.

### Data Availability

The following information was supplied regarding data availability: The alignments of sequences for ITS-rDNA, psbA non-coding forward region and reverse region and the chromatogram files of ITS-rDNA region, psbA non-coding forward region and reverse region are available in the Supplemental File. Specimens can be viewed at the National Institute of Advanced Industrial Science and Technology (AIST). A complete list of the specimen accession numbers is available in Table 1.
