# Supplementary material for: Comparison of Symbiodiniaceae diversities in different members of a Palythoa species complex (Cnidaria: Anthozoa: Zoantharia)—implications for ecological adaptations to different microhabitats
Source: PeerJ. 2020 Feb 3;8:e8449. doi: 10.7717/peerj.8449 (PMC7003691; doi:10.7717/peerj.8449)
Supplement: Table S1 [file peerj-08-8449-s001.docx]

Table S1 Composition of genotypes for ITS-rDNA sequences of Simbiodiniaceae from 4 *Palythoa* species.

|  | Genotype01 | Genotype02 | Genotype03 | A21PtToKa | A27PtToKa |
| --- | --- | --- | --- | --- | --- |
| *P. tuberculosa* | 20 | 7 | 0 | 1 | 1 |
| *P.* sp. yoron | 20 | 1 | 8 | 0 | 0 |
| *P. mutuki* | 3 | 13 | 2 | 1 | 2 |
| *P.* aff. *mutuki* | 5 | 1 | 0 | 0 | 0 |
| Total | 48 | 22 | 10 | 2 | 3 |
