# Supplementary figures and images for: Comparison of Symbiodiniaceae diversities in different members of a Palythoa species complex (Cnidaria: Anthozoa: Zoantharia)—implications for ecological adaptations to different microhabitats
Source: PeerJ. 2020 Feb 3;8:e8449. doi: 10.7717/peerj.8449 (PMC7003691; doi:10.7717/peerj.8449)

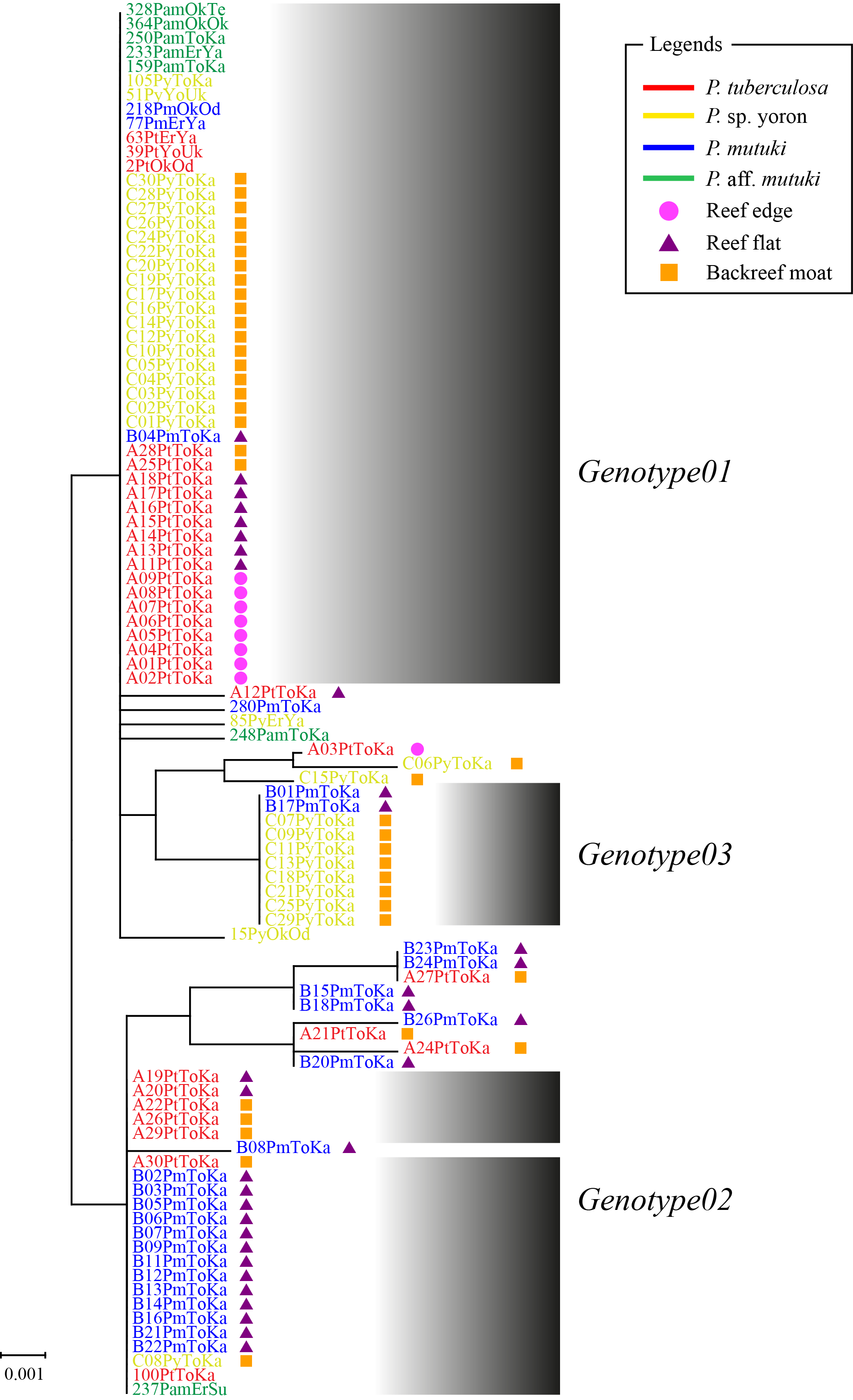

Supplement: Figure S1 — Shaded boxes represent three main genotypes occupying most sequences from four Palythoa species. Bootstrap values of maximum likelihood (ML) and neighbor joining (NJ) methods, and posterior probability (PP) are shown more than 70% for ML and NJ, and more than 0.95 for PP at the nodes, respectively. Scale bars indicate substitutions per site. Colored letters and colored diagrams represent Palythoa species and their habitats, respectively: red, P. tuberculosa; yellow, P. sp. yoron; blue, P. mutuki; green, P. aff. mutuki; circle in pink, reef edge; triangle in purple, reef flat; square in orange, backreef moat. [file peerj-08-8449-s002.png]
